# Nanovibrational stimulation inhibits osteoclastogenesis and enhances osteogenesis in co-cultures

**DOI:** 10.1038/s41598-021-02139-9

**Published:** 2021-11-23

**Authors:** Ian W. Kennedy, P. Monica Tsimbouri, Paul Campsie, Shatakshi Sood, Peter G. Childs, Stuart Reid, Peter S. Young, Dominic R. M. Meek, Carl S. Goodyear, Matthew J. Dalby

**Affiliations:** 1 Centre for the Cellular Microenvironment, Institute of Molecular, Cell and Systems Biology, College of Medical, Veterinary and Life Sciences, University of Glasgow, Glasgow, G12 8QQ UK; 2 SUPA Department of Biomedical Engineering, University of Strathclyde, Glasgow, G1 1QE UK; 3 Institute of Infection, Immunity and Inflammation, Glasgow Biomedical Research Centre, University Place, University of Glasgow, Glasgow, G12 8TA UK; 4 Department of Trauma and Orthopaedics, Queen Elizabeth University Hospital, Glasgow, G51 4TF UK

**Keywords:** Biotechnology, Cell biology, Stem cells, Medical research, Rheumatology, Nanoscience and technology, Physics

## Abstract

Models of bone remodelling could be useful in drug discovery, particularly if the model is one that replicates bone regeneration with reduction in osteoclast activity. Here we use nanovibrational stimulation to achieve this in a 3D co-culture of primary human osteoprogenitor and osteoclast progenitor cells. We show that 1000 Hz frequency, 40 nm amplitude vibration reduces osteoclast formation and activity in human mononuclear CD14^+^ blood cells. Additionally, this nanoscale vibration both enhances osteogenesis and reduces osteoclastogenesis in a co-culture of primary human bone marrow stromal cells and bone marrow hematopoietic cells. Further, we use metabolomics to identify Akt (protein kinase C) as a potential mediator. Akt is known to be involved in bone differentiation via transforming growth factor beta 1 (TGFβ1) and bone morphogenetic protein 2 (BMP2) and it has been implicated in reduced osteoclast activity via Guanine nucleotide-binding protein subunit α13 (Gα13). With further validation, our nanovibrational bioreactor could be used to help provide humanised 3D models for drug screening.

## Introduction

Osteoporosis represents a large unmet clinical need where normal bone homeostasis is disrupted, leading to reduced bone density and increased fragility^1^. This has major detrimental effects on the quality of life of an increasingly ageing population. Osteoporosis disproportionately affects women^1–3^ and fragility fractures arising from osteoporosis are a large socioeconomic burden, with an estimated cost of £4.4 billion per annum in the UK and $20 billion per annum in the US^3,4^.

Approximately 10% of total bone mass of an adult is remodelled each year, and varies by anatomical location^5^. Osteoporosis occurs due to a breakdown in the normal balanced, bone homeostasis process of formation and resorption. Treatment focuses on the use of agents such as bisphosphonates or denosumab to reduce osteoclast-related bone resorption^6^. However, more consideration needs to be directed to the mesenchymal derived osteoblast population given that it is bone formation that is reduced and limiting osteoclast formation is only slowing the disease rather than reversing it^7^. It is notable that targeting osteogenesis will also influence osteoclast regulation. This is because osteoblastic cells signal to macrophages to fuse and form osteoclasts via receptor activator of nuclear factor κB ligand (RANKL) and macrophage-colony stimulating factor (M-CSF), or prevent fusion by expression of the RANKL decoy receptor osteoprotegrin (OPG), ultimately providing homeostatic control^8^.

Due to the co-dependence and dynamic balance between bone forming and bone resorbing cells, reliance on overly simplified mono-culture cell lines, for example MG63 and RAW264.7 macrophage derived osteoclasts, may not be appropriate for use in drug discovery pipelines. This can further slow the development of new therapeutics^9^. Non-human animal models for osteoporosis are useful but, again, limited. Well used models do exist, for example, ovariectomy to model postmenopausal osteoporosis and sciatic neurectomy to model disuse osteoporosis^10^. However, rodents are the most common model and they lack the Haversian remodelling system found in humans^11^. Furthermore, only humans and primates naturally suffer from osteoporosis^11^. Thus, improved co-cultures based on human cells could be a useful tool in osteoporosis and bone homeostasis research.

To meet this need, co-cultures are being developed. Most co-cultures are based on seeding both osteoblastic and osteoclastic cell simultaneously but are generally established using murine cells or immortalised cell lines rather than primary human cells that more accurately reflect the in vivo human phenotype^12–16^. We have previously reported a simple co-culture system comprising of primary bone marrow stromal cells (BMSCs) and bone marrow hematopoietic cells (BMHCs) where osteogenic and osteoclastic development can be observed and their interactions studied^17,18^. This co-culture system was employed in this new study to test the hypothesis that nanovibrations can be used to simultaneously drive osteogenesis and reduce osteoclastogenesis.

We have recently reported a nanovibrational bioreactor that delivers 30–40 nm vertical displacements to cell cultures at 1000 Hz^19–21^. This nanomechanical stimulus drives osteospecific differentiation of BMSCs in 2D and 3D (hydrogel) cultures without need for factors such as dexamethasone, or bone morphogenetic protein 2 (BMP-2), a potent osteoinducer used in clinical practice^19–21^. In this new study, we reproduce these conditions reported as being optimal for osteogenesis^21,22^. The technique could be beneficial in helping understand osteogenesis without recourse to chemical induction and has already illustrated the roles of mechanotransductive cation channels such as TRPV1 (transient receptor potential cation channel subfamily V member 1) and piezo 1 and 2^20^. Additionally, nanovibration has been found to produce therapeutic levels of ROS, inflammation and balancing pathways^22^.

To test the effect of nanovibration, we utilised an osteoclast-forming monoculture (primary human CD14^+^ cells isolated from peripheral blood mononuclear cells, PBMCs) and our primary human BMSC/BMHC co-culture. In addition, we performed analysis in 2D and in 3D using collagen hydrogels in acknowledgement that bone is a 3D tissue.

### The nanovibrational bioreactor

**Figure 1:**
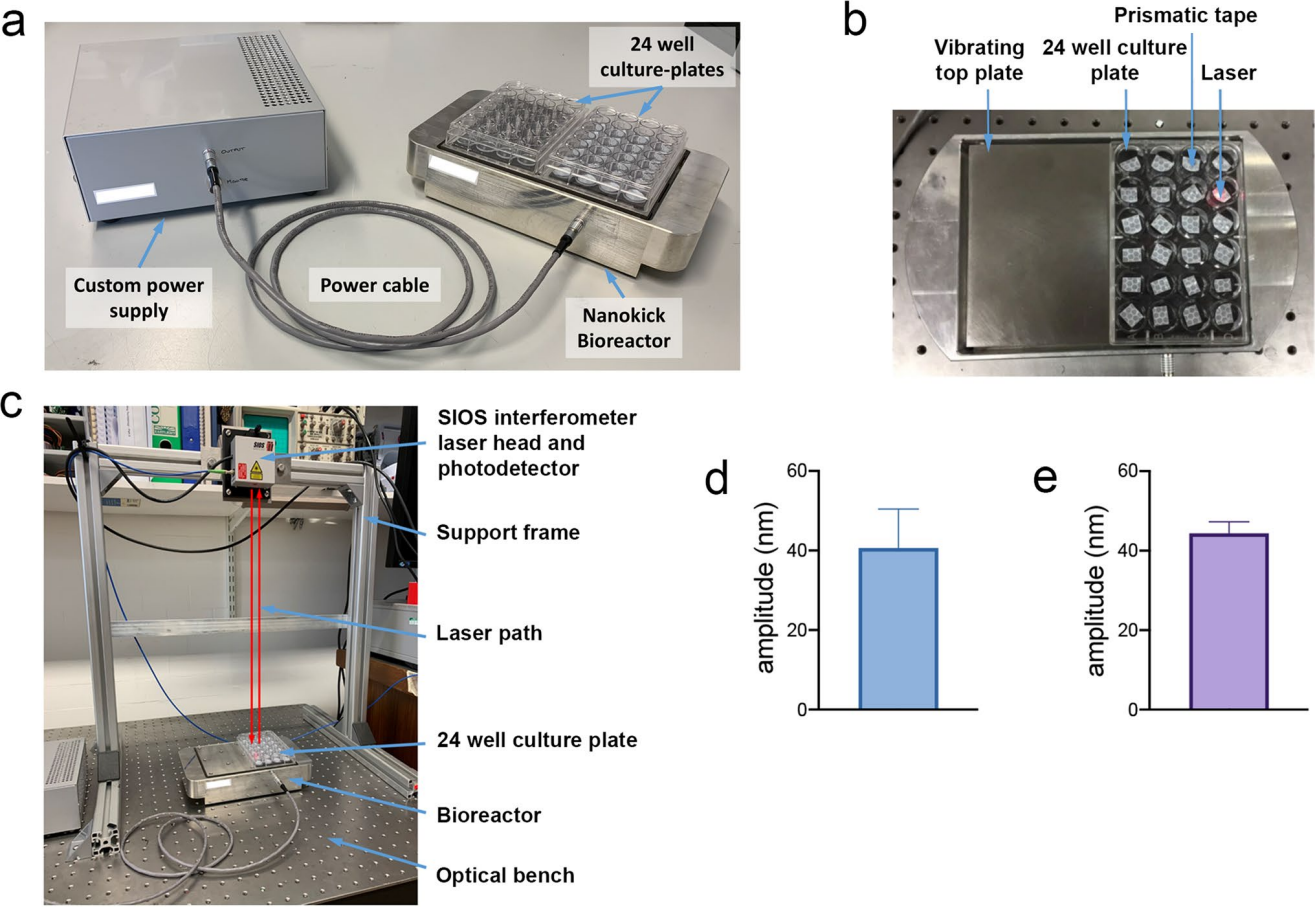
Nanovibrational stimulation setup and measurement. (**a**) Nanovibrational bioreactor and power supply. (**b**) 24 well cell culture plate attached to the bioreactor using a magnetic sheet. Reflective prismatic tape is placed in the wells (or on top of gels) so that the interferometer laser is reflected back to the detector. (**c**) Interferometer measuring bioreactor vibrations in the 24 well plate. (**d**) 2D nanovibrations in two 24 well plates. No cells were present during measurement. Measurements were taken in triplicate from each well, giving an average displacement of 40.6 nm at 1000 Hz frequency. (**e**) 3D (collagen gel) nanovibrations in a 24 well plate giving an average displacement of 44.4 nm at 1000 Hz frequency.

The bioreactor uses the reverse piezoelectric effect to produce mechanical expansions from applied voltages. Piezo actuators are attached to an aluminium base block; this mass ensures that the expansion is upwards, into the cell culture. The piezo ceramics are then glued/bolted to a ferrous top plate. This allows attachment of cell culture plastics with soft magnets. The magnets are attached to the base of the culture plates and then magnetically coupled to the bioreactor top plate. This allows easy removal of the cultureware for ease of maintenance (e.g. media changes). A power supply is used to deliver the 1000 Hz sine wave signal with a pre-determined voltage to achieve an expansion (Fig. 1a)^21^.

Laser interferometry was used to measure the nanometric displacements in 2D and 3D (collagen hydrogel) culture. To achieve this, prismatic tape was placed into the wells or on top of collagen hydrogels (0.8 mg/ml rat tail collagen) cast into the wells of 24 well plates (Fig. 1b,c)^22,23^. The collagen has low stiffness, E =  ~ 25 Pa measured by parallel plate rheology; well below that required to stimulate osteogenesis of MSCs (30–40 kPa)^24^. This ensures that while the gel is biocompatible, it is the nanovibrations that drive any osteogenesis. In 2D conditions with two 24 well plates magnetically coupled to the bioreactor top plate, an average displacement of 40.6 nm was noted (Fig. 1d). In 3D conditions with a single 24 well plate magnetically coupled, an average displacement of 44.4 nm was noted (Fig. 1e; please note that individual well vibrations are presented in Supplementary Fig. 1). Thus, we establish that the bioreactor can generate precise nanoscale vibrations in both 2D and 3D culture systems. We note that within the confines of a cell culture plate well water is incompressible^25^ and so acts as a solid object when vibrated. Equally, hydrogels, such as collagen gels, are mainly water and also transfer the mechanical motion of the vibration with little alteration of amplitude.

### Nanovibration inhibits osteoclast differentiation in CD14^+^ monoculture

**Figure 2:**
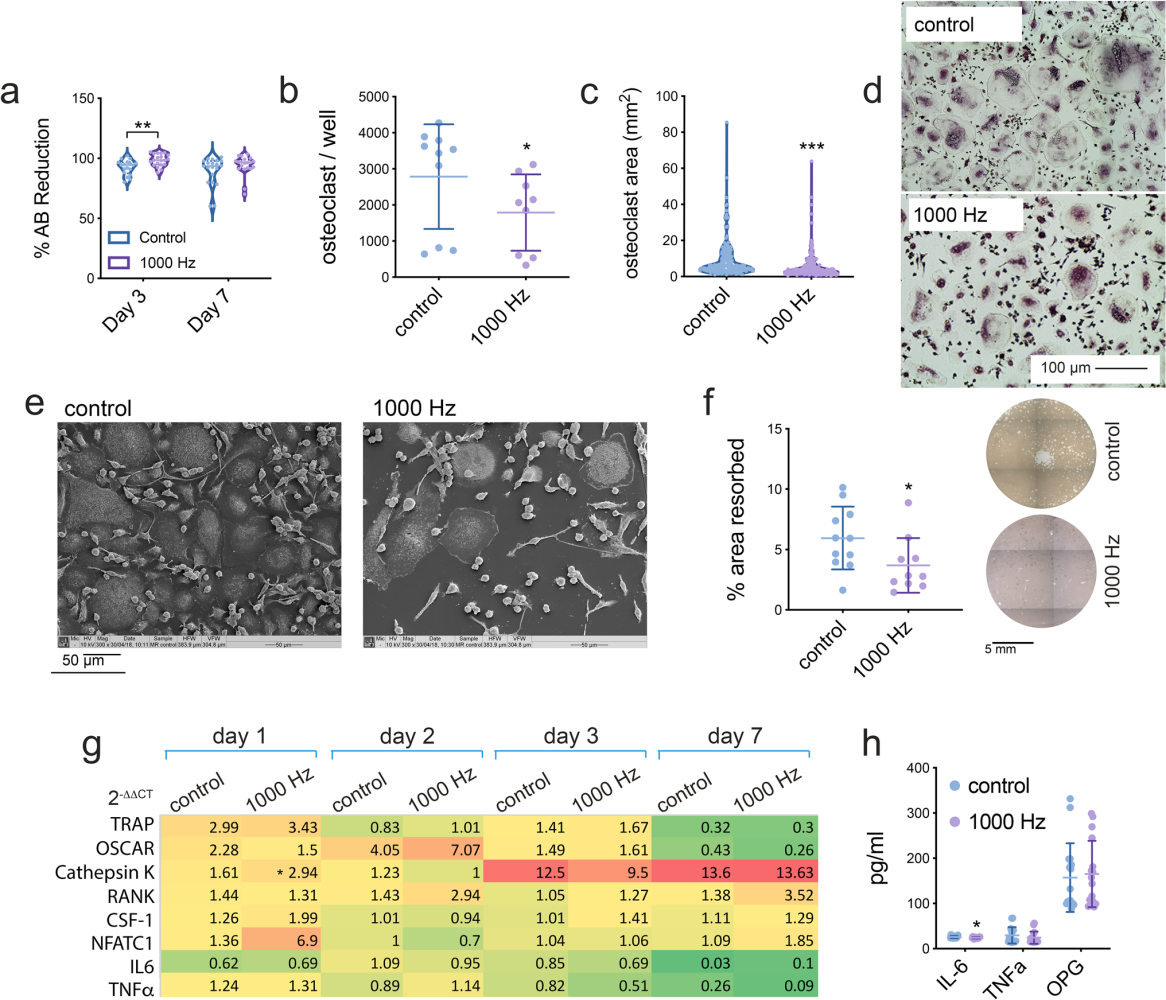
Osteoclast response to nanovibrational stimulation in 2D. (**a**) No detrimental effect on CD14^+^ cell viability was seen, as measured by Alamar blue (violin plots of individual data points, n = (d = 3, r = 3), statistics by t-test where *p < 0.05). However, (**b**) following 7 days of nanovibrational stimulation numbers of fused, multinucleate osteoclasts observed by TRAP staining were reduced (mean ± SD of individual data points, n = (d = 3, r = 3), statistics by t-test where *p < 0.05). Similarly, the mean osteoclast area (**c**) was also reduced with nanovibrational stimulation (violin plots of individual data points, n = (d = 3, r = 3), statistics by t-test where ***p < 0.001) after 7 days of culture; (**d**) typical TRAP staining of both control and 1000 Hz stimulated samples imaged at 10 × magnification. (**e**) SEM images at day 7 showing less osteoclasts were present following nanovibrational stimulation. (f) Resorption assay at day 7 showing less osteoclast activity following nanovibrational stimulation (mean ± SD of individual data points, n = (d = 3, r = 3), statistics by t-test where *p < 0.05). (**g**) qPCR for nanovibrated vs control CD14^+^ cells for transcripts related to osteoclastogenesis and inflammation. A trend towards repression of these genes in the nanovibrated cultures was observed (n = (d = 1–3, r = 3), statistics by t-test where *p < 0.05); full qPCR data is presented in Supplementary Fig. 2. (**h**) At the protein level, IL-6 was seen to be repressed (mean ± SD of individual data points, n = (d = 3, r = 4), statistics by t-test where *p < 0.05). Together, the data indicates a reduction in osteoclast forming activity of CD14^+^ blood mononuclear cells with nanovibrational stimulation.

1 × 10^6^ CD14^+^ cells isolated from PMBCs obtained from buffy coats were cultured in 2D culture in 24 well plates with 40 nm nanovibrational stimulation for up to 7 days. Non-stimulated controls were also used and all cultures (stimulated and control) were supplemented with 25 ng/ml of human M-CSF and 25 ng/ml of human RANKL in order to permit monocyte fusion and osteoclastogenesis. Alamar blue reduction was used to infer viability and showed that nanovibrational culture had no detrimental effect on the CD14^+^ cultures (Fig. 2a). The number of multinucleated osteoclasts per well after 7 days of culture was reduced (Fig. 2b), as was the average size of the osteoclasts formed (Fig. 2c). Figure 2d shows typical TRAP (tartrate resistant acid phosphatase, a marker of osteoclast formation)^13^ images used in quantitative analysis (control top, 1000 Hz stimulated bottom). Together, this infers that the monocytes seeded into the nanovibrational cultures remain viable but undergo fusion into osteoclasts less frequently. SEM (scanning electron microscopy) imaging at day 7 reflected this quantitative data showing a reduction in the number of the large osteoclasts (Fig. 2e). To study osteoclast activity, standard 24 well plates were replaced with 24 well Osteo Assay surface plates and percentage area resorbed measured after 7 days of culture. With nanovibrational stimulation, significantly less resorption was observed (Fig. 2f). The reduction in resorption was similar to that of the osteoclast cell count (approximately 30% for both resorption and cell numbers) which suggests the number of osteoclasts rather than their ability to resorb has been primarily affected. This is important, given the requirement of functional osteoclasts for normal bone remodelling and homeostasis^8^.

Looking at 2D qPCR transcriptional data for the CD14^+^ cultures, genes involved is osteoclast formation—TRAP and OSCAR (osteoclast-associated receptor)^26^ had a slight trend towards increased up-regulation with nanovibrational stimulation at day 2 and 3 (Fig. 2g). However, these changes did not reach statistical significance; an increase in cathepsin K at day 1 in the nanovibrational group was the only significant difference observed. Further, inflammatory transcripts IL-6 (interleukin 6), TNFα (tumour necrosis factor alpha)^27^ and NFATc1 (nuclear factor of activated T-cells, cytoplasmic 1) did not change between control and nanovibrated cultures (Fig. 2g). By day 7, all transcripts, apart from cathepsin K, were strongly repressed (Fig. 2g) across both control and nanovibrated cultures. It is perhaps to be expected that no change in expression of the RANKL decoy receptor and negative mediator of osteoclast formation, osteoprotegrin (OPG)^13^ was noted at any time point, as there were no osteoblast forming cells in culture. However, we note that OPG can be expressed in osteoclast monocultures as part of a self-regulatory mechanism, potentially inducing apoptosis^28^. Looking at protein expression of inflammatory mediators IL-6 and TNFα, and osteoclast inhibitor OPG by ELISA (enzyme-linked immunosorbent assay), very little change was seen after 3 days of culture; only expression of IL-6 was seen to be reduced by nanovibrational culture (Fig. 2h).

The qPCR data suggests that the monocytes follow normal gene expression patterns for both control and nanovibrated cultures and that there is no remarkable difference in expression. In order to assess if other pathways could be inferred, we used untargeted metabolomics after 3 days of nanovibrational culture. Ingenuity pathway analysis, a well curated literature-based pathway building software, was used for bioinformatics. Using molecular pathway prediction, the most changed network was around NFκB (nuclear factor kappa-light-chain-enhancer of activated B cells), predicting down-regulation with nanovibrational stimulation (Supplementary Fig. 3). NFκB is known to activate osteoclast differentiation factors c-Fos and NFATc1 (Nuclear factor of activated T-cells, cytoplasmic 1) in response to RANKL and is required for terminal osteoclast differentiation. While this seems a sensible target, we note that no activation of NFATc1 in either control of nanovibrated samples was noted in Fig. 2g and so further analysis is required^29,30^.

Together these data shows an inhibitory effect of nanovibration on osteoclast differentiation and function. These results are in agreement with previous studies that have shown slowed bone resorption with 45 Hz whole body vibration^31^ and that low-intensity pulsed ultrasound at 1.5 MHz inhibits RANKL induced osteoclast formation^32^. However, looking at standard markers of osteoclast phenotype progression, only subtle changes were noted.

### Up-regulation of osteogenesis and inhibition of osteoclastogenesis in BMSC/BMHC co-culture

For the co-culture, wells of 24 well culture plates were flooded with 3 × 10^4^ cells/ml of BMSCs in 1 ml culture media. BMSCs were isolated from human bone marrow following Ficoll gradient selection and then culturing the cells on tissue culture plates for 3 days. The adherent cells comprise the whole stromal fraction containing osteoprogenitor and mesenchymal stem cells. Concurrently, the non-adherent BMHC culture was maintained in T75 flasks until cells, presumed to be monocytes, started to adhere. At 7 days of BMSC culture, these osteoclast progenitor cells were added in to the 24 well plate at 1.2 × 10^5^ cells/ml during media change with 1 ml of culture media. The addition of the osteoclast progenitor cells was considered day 0 of the co-culture. No supplement of MCSF or RANKL was used as the BMSCs stimulate osteoclast fusion from macrophages in this culture system. Longer time points (i.e., days 7, 14, 21 and 28) than those used in the CD14^+^ culture were used to account for the lack of supplementary cytokine (and to allow sufficient time to observe osteogenesis).

**Figure 3:**
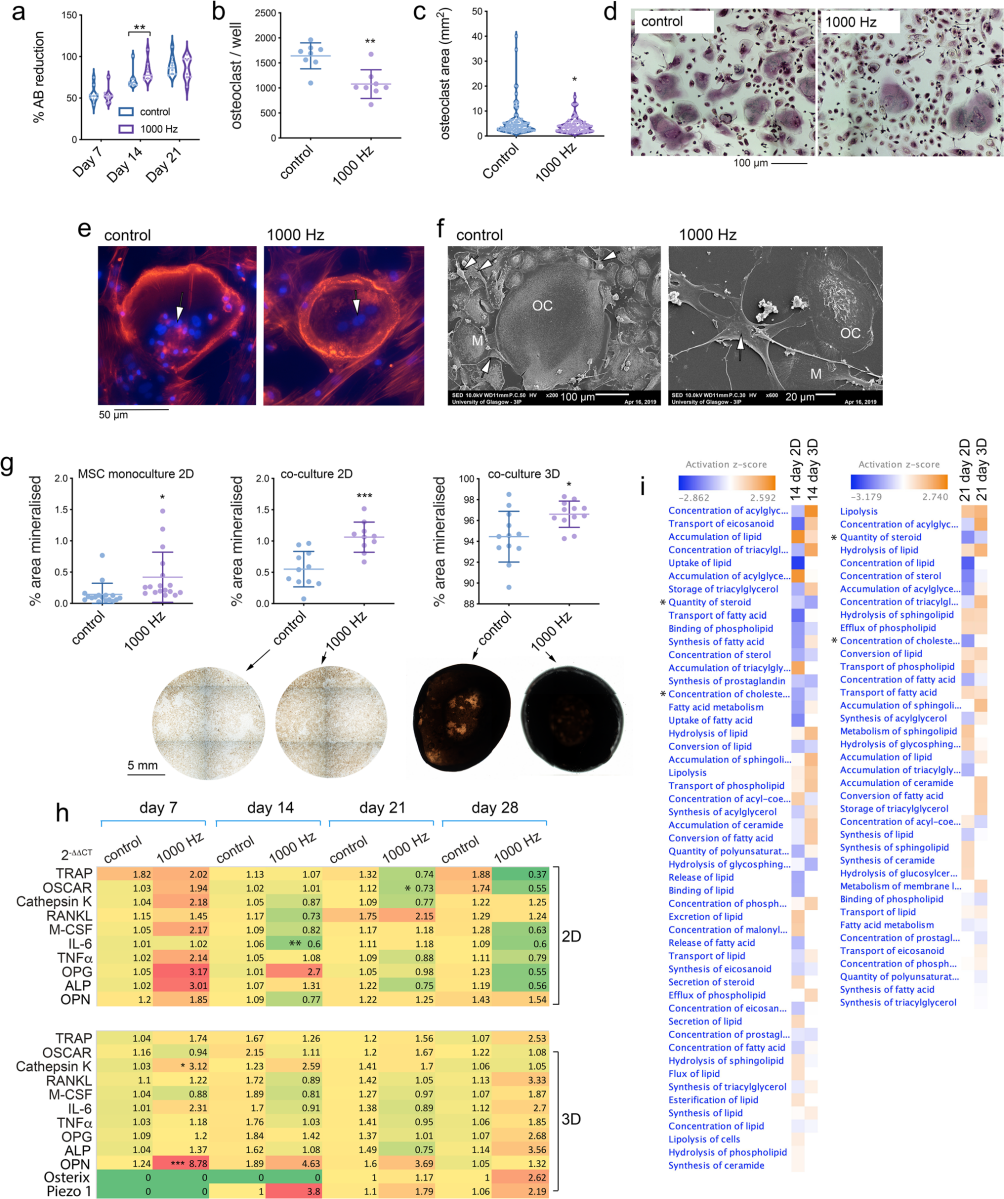
BMSC and BMHC co-culture in 2D and 3D. (**a**) No detrimental effect on cell viability, as measured by Alamar blue, was seen (violin plots of individual data points, n = (d = 3, r = 3), statistics by t-test where **p < 0.01). Using TRAP stain to identify osteoclasts after 28 days of co-culture, (**b**) the number of osteoclasts was seen to reduce (mean ± SD of individual data points, n = (d = 3, r = 3), statistics by t-test where **p < 0.01) and (**c**) area of osteoclasts was decreased with nanovibrational stimulation (violin plots of individual data points, n = (d = 3, r = 3), statistics by t-test where *p < 0.05). (**d**) Typical TRAP stain of both control and 1000 Hz stimulated samples imaged at 10 × magnification. (**e**) Actin/DAPI immunofluorescence after 28 days of culture showed that osteoclast cells identified by multiple nuclei and by actin rings tended to have fewer nuclei following nanovibrational stimulation. Arrows indicate the multiple nuclei of each cell. (**f**) SEM images after 28 days of culture showed that while many osteoclasts could be seen in control co-cultures, fewer were observed, along with better spread BMSCs, following nanovibrational stimulation. Arrows indicate BMSCs; M = macrophage; OC = osteoclast. (**g**) Looking at osteogenesis after 28 days of culture using von Kossa staining in 2D BMSC monoculture, 2D co-culture and 3D co-culture, osteogenesis was enhanced in all conditions with nanovibrational stimulation (mean ± SD of individual data points, n = (d = 3, r = 4–5), statistics by t-test where *p < 0.05, ***p < 0.001); typical von-Kossa images from the co-cultures are shown below their corresponding graphs (2D = left, 3D = right). (**h**) qPCR for nanovibrated vs control 2D (top) and 3D (bottom) co-cultures for transcripts related to osteoclastogenesis, inflammation and osteogenesis showing a trend towards initial activation and then repression of osteoclast-related genes and activation of osteoblast related genes for nanovibrated cultures (n = (d = 1–4, r = 3–4), statistics by t-test where *p < 0.05, **p < 0.01 and ***p < 0.001). Together, the data indicates reduction in osteoclast forming activity and increase in osteoblast forming activity of the co-cultures in both 2D and 3D with nanovibrational stimulation. Full qPCR data is presented in Supplementary Figs. 4 and 5. (**i**) Untargeted metabolomic analysis for 2D and 3D co-culture. Lipid-based pathways were upregulated, particularly at day 14 in the 3D culture; steroid and cholesterol pathways are indicated by *. This suggests that cell growth and differentiation is more energetically demanding in 3D culture compared to 2D culture (n = 3).

Looking at viability at days 7, 14 and 21 using Alamar blue, comparable reduction levels were seen for control and nanovibrated cultures (Fig. 3a). At 28 days of co-culture, TRAP staining was used to identify osteoclasts and it was seen that number of osteoclasts and size of osteoclasts (as a measure of fusion) decreased with nanovibrational culture (Fig. 3b–d shows typical TRAP images for control (left) and 1000 Hz stimulated (right) cultures). Furthermore, at day 28 of co-culture, actin cytoskeleton/DAPI and SEM images were taken. In line with quantitative data on size (Fig. 3b,c), osteoclasts (denoted by multinuclei and typical actin ring) tended to have fewer nuclei per cell (Fig. 3e) and tended to be smaller (Fig. 3f). SEM images clearly showed the macrophage, osteoclast and BMSC co-culture (Fig. 3f).

Considering osteogenesis of the BMSCs in co-culture, von Kossa stain for mineralization was employed at day 28 of co-culture. First looking at 2D BMSC monoculture, enhanced mineralization was seen as expected (Fig. 3g)^20^. For the 2D co-culture, mineralization was seen to be highly significantly enhanced (Fig. 3g). Finally, for the 3D co-culture within collagen gels BMSC mineralization was seen to be significantly increased. This shows, for the first time, that nanovibrational osteogenesis is maintained in co-culture conditions.

Next, qPCR was used to measure transcripts related to the BMHC (TRAP, OSCAR, Cathepsin K, IL6, TNFα and OPG) and BMSC (RANKL, M-CSF, alkaline phosphatase (ALP), osteopontin (OPN), osterix and the mechanosensitive ion channel piezo 1, all of which relate to the osteoblast phenotype) populations in 2D and 3D co-cultures. For both 2D and 3D co-cultures, there was a trend, mainly in the osteoclast maturity transcripts, that with nanovibration, transcripts were up-regulated at day 7 compared to control cultures but then tended to become down-regulated at days 14, 21 and 28 (Fig. 3h). In 2D, this included significant reductions in IL-6 at day 14 and OSCAR at day 21 in the 2D culture. Considering osteogenic transcripts, a non-significant trend of increased osteogenic transcript expression (ALP and OPN) was noted in 2D. In 3D, the osteogenic pattern was more apparent with significant upregulations in OPN transcript expression, as well as increased trends of expression for ALP and for piezo 1, a mechanosensitive ion channel implicated in osteogenesis^20,33^. This data supports osteogenesis occurring even in co-culture where RNA from both BMSCs and BMHCs was isolated.

Lipid expression, involved in energy pathways, are regularly cited as changing with physical stimuli^34–36^. Thus, untargeted metabolomic analysis was employed to study fold change to unstimulated control between 2 and 3D nanovibrational co-cultures. At both 14 and 21 days of co-culture, it was seen that lipid-based pathways were differentially regulated in nanovibrated cultures (2D and 3D) compared to control cultures based on patterns of lipid abundance (Fig. 3i). This difference was most apparent at the earlier time point of culture (day 14). This suggests, potentially, that cell growth and differentiation is more energetically demanding in 3D culture compared to 2D culture. Further, it is known that depletions of steroids (e.g. dexamethasone) and cholesterols (e.g. cholesterol sulphate) are important in osteogenesis and this was observed in nanovibrated cultures compared to controls (Fig. 3i)^37–39^.

Further pathway analysis of untargeted metabolomics data found that metabolites feeding into protein kinase B (Akt) could be found to be differentially regulated compared to controls at both days 14 and 21 of co-culture for 2D and 3D nanostimulation, with Akt expression predicted to be up-regulated at day 14 and down-regulated at day 21 (Supplementary Fig. 6). Akt is known to be important in osteogenesis and osteoclast formation^40^.

## Discussion

We report on a mechanical bioreactor that provides nanoscale stimulation to osteoblastic cells while reducing osteoclast formation in appropriate co-culture conditions. Such a methodology could find use in several ways: as a tissue engineering approach, as a direct therapy and as a drug discovery platform.

Bone is the second most commonly transplanted tissue after blood^41^. The current gold-standard is autologous bone graft (i.e., harvested from the same individual) due to its osteogenic, osteoconductive and osteoinductive properties^41^. However, allograft—in such forms as demineralised bone matrix, morcellised cancellous chips or whole bone segments—are also frequently utilised^41^. There are well-evidenced disadvantages to the current graft options, including donor site pain and complications^42,43^, and off-target effects in the case of BMP-2^44,45^. Tissue engineering approaches, such as drug-free 3D bone differentiation, as we demonstrate here, represent an attractive option to overcome the aforementioned challenges and as such have been an area of significant interest in the scientific community^46^.

As well as stimulating osteogenesis of BMSCs, we demonstrate, for the first time, reduction of osteoclast formation and activity in 2D and 3D co-cultures. This is important as the interplay between osteoblasts and osteoclasts is crucial to normal bone development, homeostasis and remodelling after injury. Any imbalance in this dynamic, particularly with regard to osteoclast function, can lead to pathological conditions, such as osteoporosis^8,47^ and osteopetrosis^48,49^. With the ageing population, in whom degenerative and pathological conditions such as osteoporosis are more prolific, the demand for innovation has never been higher^46,50, 51^. As discussed, application of whole body vibration^31^ and low-intensity pulsed ultrasound inhibit osteoclastogenesis^32^. There are also indications that these vibration approaches may aid bone regeneration^52–55^. However, it is difficult to determine from in vivo studies whether it is muscle tone, bone regeneration or osteoclast formation that is altered, and in vitro studies that look at only one cell type in isolation are limited as a consequence^52–55^. Our approach allows us to understand that both bone formation and osteoclast fusion and activity are effected at the intermediate, 1000 Hz, frequency. This allows us to envisage a direct application of nanovibration, as with whole body vibration^56^ and low-intensity pulsed ultrasound^57^, after further in vivo evaluation using technologies similar to bone conduction headphones (1000 Hz is an audible signal)^58^. Our human, 3D model of bone homeostasis where osteogenesis is enhanced and osteoclastogenesis reduced, and where potential pathways can be identified, could also be useful in researching new drugs for conditions such as osteoporosis. Further validation of the metabolomic findings could strengthen the use of this model.

## Conclusion

1000 Hz nanovibrational stimulation reduces osteoclast formation and activity in monocultures. In primary 2D and 3D human osteoprogenitor/osteoclast progenitor co-cultures, the 1000 Hz stimulation leads to enhanced osteogenesis and reduced osteoclast formation. This provides a model of bone remodelling where bone formation is dominant to loss and use of untargeted metabolomics allows identification of controlling pathways.

## Materials and methods

This work has been approved by the NHS research Scotland (NRS) Greater Glasgow and Clyde Bio-repository ethics committee. All methods were carried out in accordance with relevant guidelines and regulations. Informed consent was obtained from all subjects who donated tissue samples.

### CD14^+^ culture

Buffy coats were received from the Scottish National Blood Transfusion Service. Magnetic selection was performed using EasySep Human CD14 positive selection kit II (product no. 17858, Stemcell Technologies, Cambridge, UK. The cells were re-suspended at 1 × 10^6^ cells/ml in α-MEM (alpha minimum essential media) supplemented with 10% FBS, 0.02 mM l-glutamine, 10 U/ml Penicillin, and 0.1 μg/ml Streptomycin (all components from Sigma-Aldrich, Dorset, UK). Following suspension at 1 × 10^6^ cells/ml, 25 ng/ml of recombinant human M-CSF (product no. 300-25, Peprotech, London, UK) was added. Cells were plated in 24 well plates and incubated overnight at 37 °C and 5% CO_2_. After approximately 18 h incubation 25 ng/ml of human RANKL (product no. 310-01, Peprotech, London, UK) was added to a proportion of the wells. Those wells with M-CSF and no RANKL were used as a negative control of osteoclastogenesis.

### Co-culture

Human bone marrow was aspirated from patients undergoing elective hip and knee arthroplasty. The bone marrow aspirate was washed and centrifuged to produce a cell pellet. The cell pellet was re-suspended and overlaid on a Ficoll-Paque 1.073 (No. 17-5446-52, Sigma-Aldrich, Dorset, UK) gradient and the subsequent mononuclear interface layer aspirated and resuspended in mDMEM ((dulbecco’s Modified Eagle Medium) (Sigma-Aldrich, Dorset, UK), 10% FBS (Sigma-Aldrich, Dorset, UK), 1% Sodium Pyruvate (11 mg ml^−1^, Sigma-Aldrich, Dorset, UK), 1% Gibco MEM NEAA (non-essential amino acids, Thermo Fisher Scientific, Loughborough, UK), 2% antibiotics (6.74 U ml^−1^ penicillin–streptomycin (Sigma-Aldrich, Dorset, UK) and 0.2 μg ml^−1^ fungizone (Sigma-Aldrich, Dorset, UK)). The cells were then plated at a density of 1 × 10^6^ in a 75 cm^2^ vented cell culture flask and incubated at 37 °C with 5% humidified CO_2._ At day 3 non-adherent cells were removed and cultured separately. This non-adherent fraction contained mainly bone marrow haematopoietic cells (BMHC), macrophages and osteoclast precursors. The remaining adherent cells were assumed to be mesenchymal stromal cells (MSCs), osteoprogenitors, osteoblasts and osteocytes and were cultured until an approximately 80% confluent layer was obtained. The adherent cells were then detached and resuspended in mDMEM to a concentration of 3 × 10^4^ cells/ml. 1 ml of cell suspension/well was pipetted onto a 24 well plate. At day 7, 1 ml of BMHC suspension/well was added at a concentration of 1.2 × 10^5^/ml.

### 3D cell culture technique

Cells were isolated as discussed. Type 1 rat collagen gel was then prepared by adding sodium hydroxide (NaOH), 10% FBS and 10 × DMEM. Cells were added to the gel and further NaOH was added drop-wise while agitating the mix until the colour changed from yellow to pink, indicating the correct pH had been achieved. 1 ml of gel/well was pipetted on to a 24 well plate. 1 ml of media ± growth factors was added on top of the gels after 30 min.

### TRAP staining

Cells were fixed after 7 days for the CD14^+^ culture and 28 days for the co-culture. TRAP (No. 387A, Sigma-Aldrich, Dorset, UK) was used as per manufacturer’s instructions. Samples were then washed and air dried. Digital images of the entire wells were acquired. Osteoclasts were identified as TRAP positive cells with ≥ 3 nuclei and quantified by manual counting of both number and area.

### Immunostaining

Co-culture samples were cultured for 28 days. Medium was removed and cells were fixed with 3.7% methanol-free formaldehyde (Sigma-Aldrich, Dorset, UK) for 10 min at room temperature. Samples were then washed twice with 1 × PBS (phosphate buffer saline) and permeabilised with 0.1% Triton x-100. Rhodamine-conjugated phalloidin (No. R415, Thermo Fisher Scientific, Loughborough, UK) was added for an hour in the dark (1:300 in 1% BSA (bovine serum albumin)/1 × PBS). Samples were washed before adding DAPI and viewing the samples with a fluorescent microscope to visualise the actin rings and nuclei using an EVOS® FL Auto 2 Cell Imaging System.

### Von Kossa staining

Samples were cultured for 28 days then fixed with 4% formaldehyde solution. 5% silver nitrate was added before exposing to UV light for 30 min. Samples were washed, then 5% sodium thiosulphate added for 10 min. A further wash was performed before counterstaining with 0.1% nuclear fast red. A final wash with deionized water was performed then a further rinse with 70% ethanol. Images of the entire well were obtained and the percentage surface area mineralised calculated.

### Scanning electron microscopy

Cells were fixed with buffered glutaraldehyde fixative for 1 h at 4 °C then rinsed with 0.1 M sodium cacodylate. Postfix in 1% osmium tetroxide for 1 h at room temperature was then performed. A dehydration process was carried out using an ethanol series. Hexamethyl-disilazane was then applied to samples and left overnight for drying. An 18 nm gold palladium coating was overlaid on the sample surface using a Polaron SC515 SEM Coating System. Finally, samples were attached to aluminium stubs and analysed on a Carl Zeiss Sigma variable-pressure analytical SEM^20^.

### Alamar Blue assay

Cell viability was assessed at defined time points using an Alamar Blue resazurin (No. DAL1025, Thermo Fisher Scientific, Loughborough, UK) assay according to the manufacturer’s instructions. A 10% solution was produced. Media was removed from the wells and 500 µl of 10% Alamar Blue added. Both the stimulated and control samples were then incubated at 37 °C for 4 h in the dark. 150 µl/well of 10% Alamar Blue was then transferred in triplicate to a 96 well plate. The remainder of 10% Alamar Blue was discarded before washing with HEPES saline and adding fresh media. The absorbance was measured at 570 nm and 600 nm using a Multiskan FC Microplate Photometer^20^. The percentage of Alamar Blue reduction was calculated using the formula:

$$\% \, \;{\text{reduction }}\;{\text{of }}\;{\text{Alamar}}\;{\text{ Blue }} = \, \left( {\left( {{\text{O2 }} \times {\text{ A1}}} \right) \, {-} \, \left( {{\text{O1 }} \times {\text{ A2}}} \right) \, / \, \left( {{\text{R1 }} \times {\text{ N2}}} \right) \, - \, \left( {{\text{R2 }} \times {\text{ N1}}} \right)} \right) \, \times { 1}00$$

O1 and O2 = the molar extinction coefficients of oxidised Alamar Blue at wavelengths 570 nm and 600 nm respectively. R1 and R2 = molar extinction coefficients of the reduced Alamar Blue at wavelengths 570 nm and 600 nm respectively. A1 and A2 = observed absorbance readings for test wells at wavelengths 570 nm and 600 nm respectively. N1 and N2 = observed absorbance readings for the negative control wells at wavelengths 570 nm and 600 nm respectively^20^.

### RT-qPCR

Following culture for defined time points, RNA in the 2D culture was extracted using the RNeasy Micro Kit (No. 74004, QIAGEN, Manchester, UK) according to the manufacturer’s instructions. An alternative technique for RNA extraction was used for the 3D culture where gels were disrupted and TRIzol (No. 15596026, Thermo Fisher Scientific, Loughborough, UK) added for 10 min. Samples were centrifuged and the RNA in solution transferred to a clean eppendorf. 100 µl chloroform was added for 3 min. Samples were centrifuged and the upper aqueous phase transferred to a clean tube. 250 µl isopropanol was then mixed in to samples. The eppendorfs were incubated at − 20 °C for 1 h and centrifuged. The supernatant was discarded without disrupting the pellet. The RNA pellet was washed twice with 70% ethanol and centrifuged. The ethanol was removed and the tube air-dried. The pellet was re-suspended in 20 µl RNase-free water and incubated at 60 °C for 10 min. This final incubation facilitates dissolution of the RNA pellet into solution, which otherwise would not be accessible. Heating to 60 °C for 10 min for this purpose follows the manufacturer’s instruction. In both 2D and 3D cultures, nucleic acid quantification was performed using a Nanodrop 1000 spectrophotometer. Reading distilled water absorbance acted as a blank, and the samples were read at 260 nm to give quantification and the ratio of 260/280 used for purity estimation. 100–300 ng of RNA was used to generate cDNA using QuantiTect Reverse Transcription Kit (No. 205311, QIAGEN, Manchester, UK). cDNA was further diluted to a concentration of 5 ng/µl and stored at − 20 °C. RT-qPCR (reverse transcription quantitative polymerase chain reaction) was performed using a SYBR green kit (No. 204143, QIAGEN, Manchester, UK). An Abi7500 thermal cycler (Thermo Fisher Scientific, Loughborough, UK) was used. The primer sequences (Table 1) for the genes were validated by dissociation curve/melt curve analysis. The GapDH (glyceraldehyde 3-phosphate dehydrogenase) housekeeping gene primer was used for normalisation. The ΔCT of each sample was calculated with normalisation against GapDH. qRT-PCR product quantification was performed using the 2^−ΔΔCt^ method.

**Table 1:** Forward and reverse primer sequences for RT-qPCR.

Target gene	Forward sequence	Reverse sequence
ALP	AGAACCCCAAAGGCTTCTTC	CTTGGCTTTTCCTTCATGGT
Cathepsin-K	GCCAGACAACAGATTTCCATC	CAGAGCAAAGCTCACCACAG
CSF-1	TCCCAGTGATAGAGCCCAGT	CAGGGTCCAGTGAGGTGATG
IL-6	GATGAGTACAAAAGTCCTGATCCA	CTGCAGCCACTGGTTCTGT
M-CSF	GAACTGCCAGTGTAGAGGGAAT	GCTGGTCAGACAACATCTGG
NFATc1	CACCAAAGTCCTGGAGATCCCA	TTCTTCCTCCCGATGTCCGTCT
OSCAR	CCAGCTCTAGCGGGTATCTG	GACGGAGTGATGTCTGTGTGAC
Osterix	GGCAAAGCAGGCACAAAGAAAG	AATGAGTGGGAAAAGGGAGGG
OPN	AGCTGGATGACCAGAGTGCT	TGAAATTCATGGCTGTGGAA
OPG	GAAGGGCGCTACCTTGAGAT	GCAAACTGTATTTCGCTCTGG
Piezo 1	CCTGGAGAAGACTGACGGCTAC	ATGCTCCTTGGATGGTGAGTCC
RANK	GCTGTAACAAATGTGAACCAGGA	GCCTTGCCTGTATCACAAACT
RANKL	TGATTCATGTAGGAGAATTAAACAGG	GATGTGCTGTGATCCAACGA
TNF-α	CAGCCTCTTCTCCTTCCTGAT	GCCAGAGGGCTGATTAGAGA
TRAP	GGACTGAAGGGACTCCTGAAT	GGTCCCTGAGCCTTTATTCC

### ELISA

The supernatant was removed from culture wells and stored pending analysis at − 80 °C. The media was last changed 72 h prior to removal for analysis. ELISA plates were prepared according to the manufacturer’s instructions (IL-6 No. DY206, TNF-α No. DY201, OPG No. DY805, R&D Systems, Abingdon, UK). Plates were then washed and blocked with reagent diluent. A further wash was performed before adding samples and standards. The detection antibody was then added before washing. Streptavidin-HRP was added, avoiding direct light exposure. A further wash was performed. Substrate solution was added for 20 min, avoiding direct light exposure. Finally, stop solution was added. The optical density was calculated at 450 nm using a Multiskan FC Microplate Photometer. A standard curve was created with Prism v6 and the results interpolated from this.

### Osteoclast functional assessment

The CD14^+^ cell culture was seeded at a concentration of 1 × 10^6^/ml onto a 24 well Osteo Assay Surface plate (No. 3987, Corning, Flintshire, UK). RANKL was added at a concentration of 25 ng/ml after approximately 18 h incubation. After 7 days incubation, the cell layer was removed by adding 10% chlorine solution for 10 min. The wells were rinsed before leaving to dry. Images were then captured using an EVOS® FL Auto Cell Imaging System and the area of resorption calculated.

### Metabolomics

All the media was removed from the wells before washing with chilled 1 × PBS. 500 µl/well of chilled extraction solvent (1:3:1 chloroform: methanol: water) was used. Plates were then sealed with parafilm and vigorously agitated on a rotary shaker for 1 h at 4 °C. The solvent was removed from the wells and placed in an eppendorf before centrifuging at 1300 rpm for 3 min. The supernatant was removed and placed in a new eppendorf. 50 µl of each sample was placed in a separate tube to produce a pooled sample of each condition. Liquid chromatography-mass spectrometry (LC–MS) was performed using the UltiMate 3000 RSLC and Orbitrap Q-Exactive (Thermo Fisher Scientific, Loughborough, UK). The data produced by LC–MS was converted to an IDEOM file. This Excel file details the varying metabolites identified and their respective quantities. Furthermore, the metabolites are linked to the KEGG database (Kyoto Encyclopaedia of Genes and Genomes) which facilitates examination of varying chemicals and biological pathways^59^. The files were generated containing the metabolite KEGG IDs and their ratio to the specified control. These files were analysed with IPA (ingenuity pathway analysis); a programme that identifies and predicts alterations in pathways and networks. No KEGG images or pathways were presented in this study, only IPA networks.

### Statistical analysis

The appropriate statistical analyses were preformed using GraphPad Prism software (version 6). Normally distributed data were analysed with the t-test. Conversely, if the data were not normally distributed the Mann–Whitney *U* test was utilised. Statistically significant results were defined as those having a *p* value of 0.05 or less.

## Supplementary Information

Supplementary Information 1.

Supplementary Figure S1.

Supplementary Figure S2.

Supplementary Figure S3.

Supplementary Figure S4.

Supplementary Figure S5.

Supplementary Figure S6.
