# Supplementary material for: Nanovibrational stimulation inhibits osteoclastogenesis and enhances osteogenesis in co-cultures
Source: Sci Rep. 2021 Nov 23;11:22741. doi: 10.1038/s41598-021-02139-9 (PMC8611084; doi:10.1038/s41598-021-02139-9)
Supplement: Supplementary file 1 — Supplementary Information 1. [file 41598_2021_2139_MOESM1_ESM.docx]

**Supplementary data for:**

Nanovibrational stimulation inhibits osteoclastogenesis and enhances osteogenesis in co-cultures.

Ian W. Kennedy, P. Monica Tsimbouri, Paul Campsie, Shatakshi Sood, Peter G. Childs, Stuart Reid, Peter S. Young, Dominic R.M. Meek, Carl S. Goodyear, Matthew J. Dalby

***Supplementary figure 1.*** *Nanovibrational measurement. Vibrational readouts from 24 well plates showing the variance in vibration in 2D and 3D. Values are the mean of 3 readings.*

***Supplementary figure 2.*** ***Osteoclast response to nanovibrational stimulation in 2D.*** *Ingenuity pathway analysis of metabolite data after 7 days of culture inferred inhibition of NFκB signalling with nanovibration (n=3). Images produced by MJD using IPA.*

***Supplementary Figure 3.* *Metabolite network analysis implying differential Akt regulation.*** *Untargeted metabolite analysis for 2D and 3D nanovibrational cultures compared to controls at days 14 and 21 of culture all linked to Akt signalling. Akt was predicted to be up-regulated at day 14 and down-regulated at day 21 (n=(d=3,r=4)). It has been implicated in bone cell differentiation, specifically TGFβ1 (transforming growth factor beta 1) and BMP2 (bone morphogenetic protein 2) stimulated osteogenesis.^1, 2^ Disruption of Akt1 causes osteoblast apoptosis and decreased activity of the master osteoblast transcriptional regulator runt-related transcription factor 2 (RUNX2); this in turn reduces RANKL expression and prevents osteoclast fusion.^3^ Inhibition of Akt has also been potentially implicated in reduced osteoclastogenesis. Guanine nucleotide-binding protein subunit α13 (Gα13) negatively regulates osteoclast formation through increased Akt/GSK3β/NFATc1 (glycogen synthase kinase 3 beta / nuclear factor of activated T cells 1) signalling.^4^ Further, Akt inhibition rescues osteoclast hyper-activation in Gα13 deficient osteoclasts. However, M-CSF and RANKL stimulation of osteoclastogenesis stimulates Akt to mediate survival, proliferation and differentiation of osteoclast progenitors.^4, 5^ This effect was only demonstrated in the co-culture, and as such the initial implied increase in Akt signalling seen here could therefore be acting to enhance osteogenesis and moderate osteoclastogenesis. However, the metabolomics data represents a correlative association and causation has not been confirmed in this study. Images produced by MJD using IPA.*

***Supplementary references.***

1. Mukherjee, A. & Rotwein, P. Akt promotes BMP2-mediated osteoblast differentiation and bone development. *J Cell Sci* **122**, 716-726 (2009).

2. Suzuki, E. et al. Akt activation is required for TGF-beta1-induced osteoblast differentiation of MC3T3-E1 pre-osteoblasts. *PLoS ONE* **9**, e112566 (2014).

3. Kawamura, N. et al. Akt1 in osteoblasts and osteoclasts controls bone remodeling. *PLoS ONE* **2**, e1058 (2007).

4. Wu, M. et al. Galpha13 negatively controls osteoclastogenesis through inhibition of the Akt-GSK3beta-NFATc1 signalling pathway. *Nature communications* **8**, 13700 (2017).

5. Matsumoto, T. et al. Regulation of bone resorption and sealing zone formation in osteoclasts occurs through protein kinase B-mediated microtubule stabilization. *J Bone Miner Res* **28**, 1191-1202 (2013).
