# Supplementary figures and images for: Nanovibrational stimulation inhibits osteoclastogenesis and enhances osteogenesis in co-cultures
Source: Sci Rep. 2021 Nov 23;11:22741. doi: 10.1038/s41598-021-02139-9 (PMC8611084; doi:10.1038/s41598-021-02139-9)

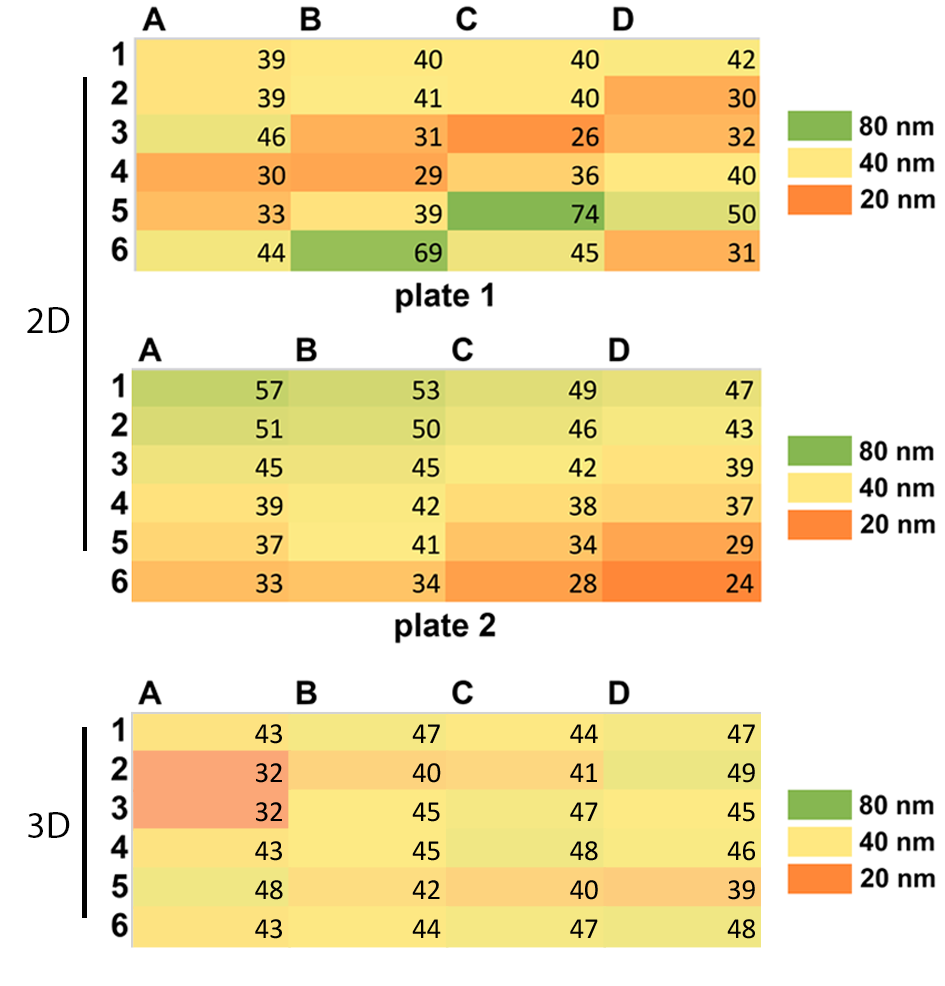

Supplement: Supplementary file 2 — Supplementary Figure S1. [file 41598_2021_2139_MOESM2_ESM.tif]

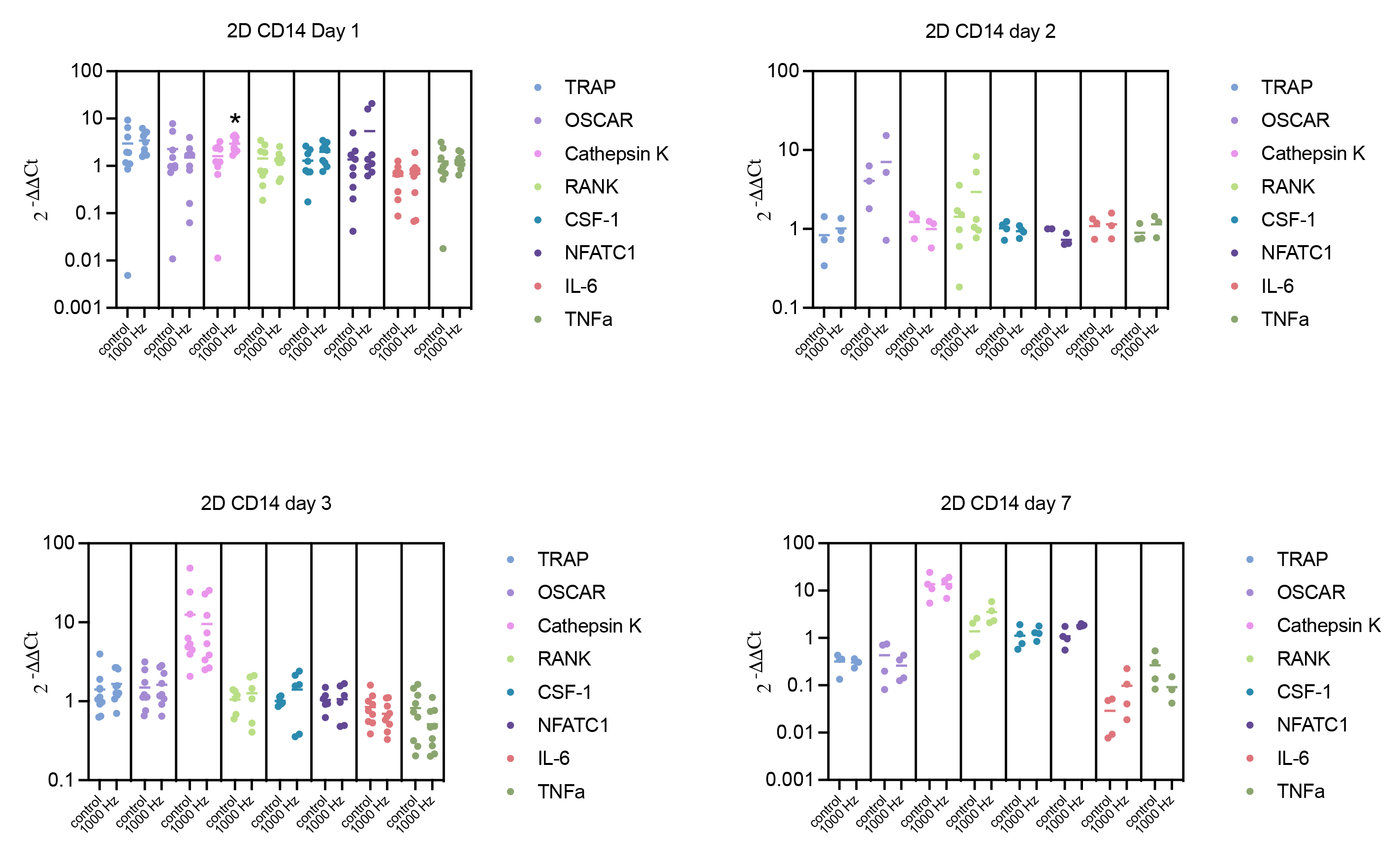

Supplement: Supplementary file 3 — Supplementary Figure S2. [file 41598_2021_2139_MOESM3_ESM.tif]

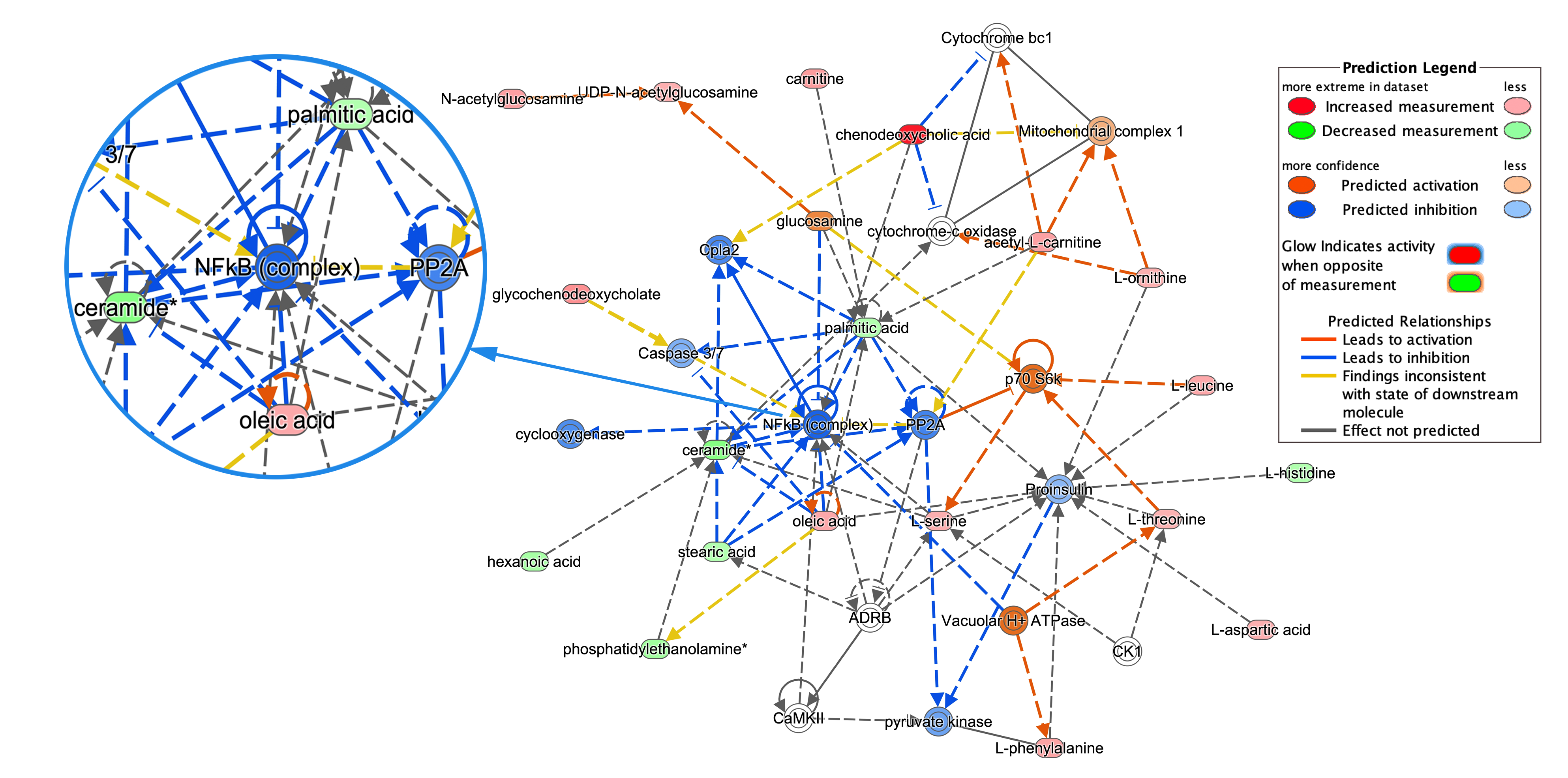

Supplement: Supplementary file 4 — Supplementary Figure S3. [file 41598_2021_2139_MOESM4_ESM.tif]

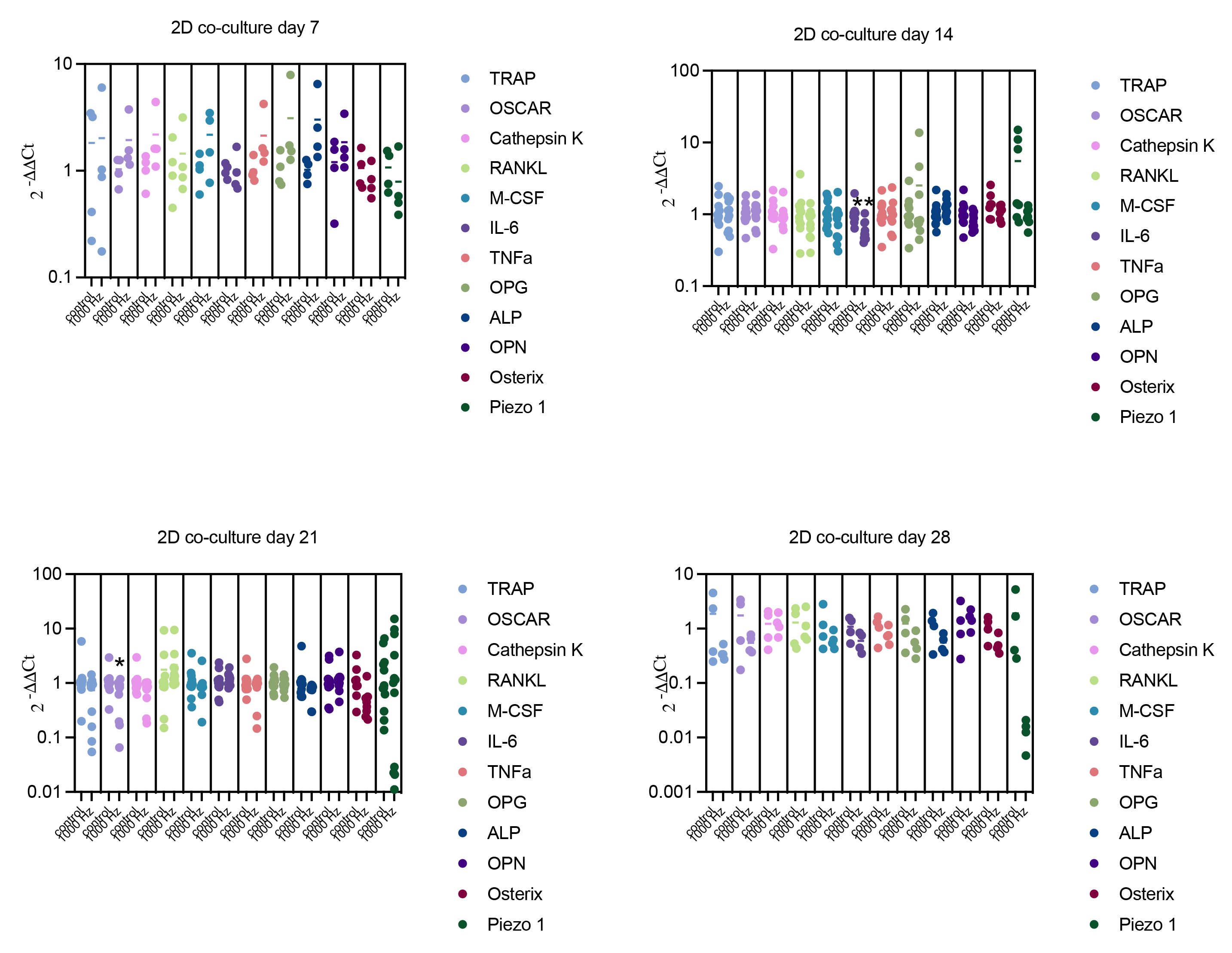

Supplement: Supplementary file 5 — Supplementary Figure S4. [file 41598_2021_2139_MOESM5_ESM.tif]

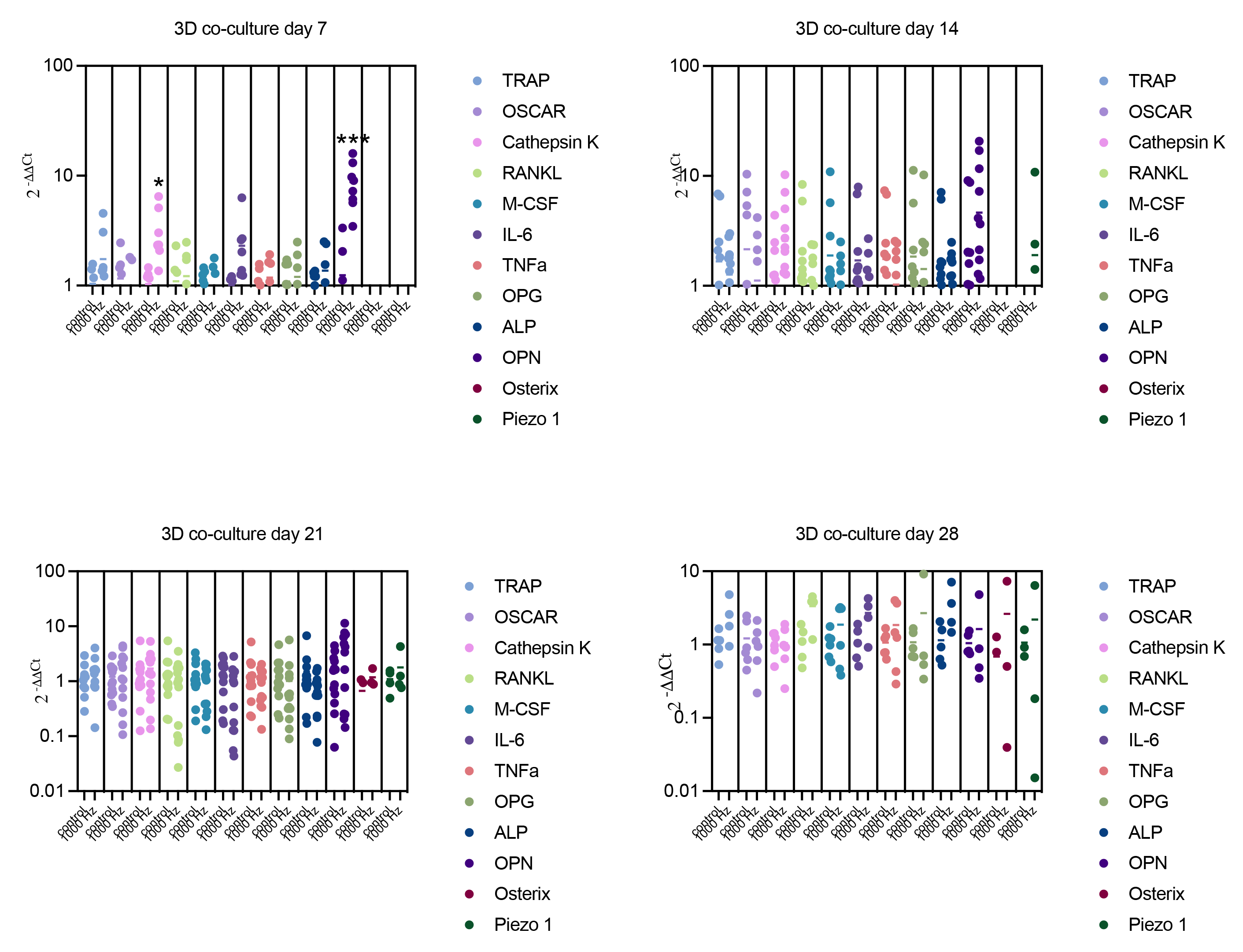

Supplement: Supplementary file 6 — Supplementary Figure S5. [file 41598_2021_2139_MOESM6_ESM.tif]

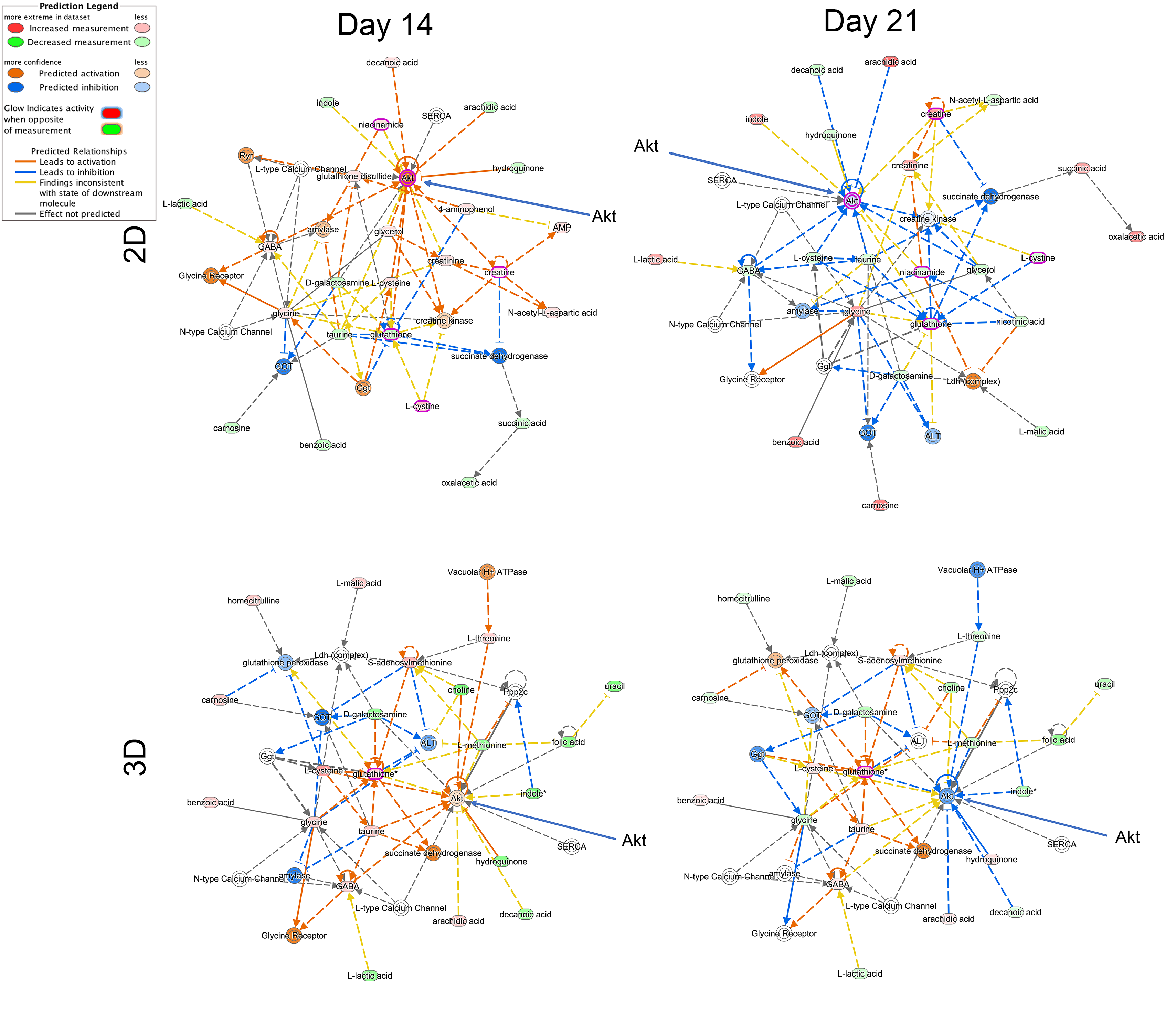

Supplement: Supplementary file 7 — Supplementary Figure S6. [file 41598_2021_2139_MOESM7_ESM.tif]
